# Marginal hazard ratio estimates in joint frailty models for heart failure trials

**DOI:** 10.1002/bimj.201800133

**Published:** 2019-06-17

**Authors:** Gerrit Toenges, Antje Jahn‐Eimermacher

**Affiliations:** ^1^ Institute of Medical Biostatistics, Epidemiology and Informatics University Medical Center of the Johannes Gutenberg University Mainz Mainz Germany; ^2^ Department of Mathematics and Natural Sciences Darmstadt University of Applied Sciences Darmstadt Germany

**Keywords:** heart failure trials, joint frailty model, least false parameter, recurrent events, unexplained heterogeneity

## Abstract

This work is motivated by clinical trials in chronic heart failure disease, where treatment has effects both on morbidity (assessed as recurrent non‐fatal hospitalisations) and on mortality (assessed as cardiovascular death, CV death). Recently, a joint frailty proportional hazards model has been proposed for these kind of efficacy outcomes to account for a potential association between the risk rates for hospital admissions and CV death. However, more often clinical trial results are presented by treatment effect estimates that have been derived from marginal proportional hazards models, that is, a Cox model for mortality and an Andersen–Gill model for recurrent hospitalisations. We show how these marginal hazard ratios and their estimates depend on the association between the risk processes, when these are actually linked by shared or dependent frailty terms. First we derive the marginal hazard ratios as a function of time. Then, applying least false parameter theory, we show that the marginal hazard ratio estimate for the hospitalisation rate depends on study duration and on parameters of the underlying joint frailty model. In particular, we identify parameters, for example the treatment effect on mortality, that determine if the marginal hazard ratio estimate for hospitalisations is smaller, equal or larger than the conditional one. How this affects rejection probabilities is further investigated in simulation studies. Our findings can be used to interpret marginal hazard ratio estimates in heart failure trials and are illustrated by the results of the CHARM‐Preserved trial (where CHARM is the ‘Candesartan in Heart failure Assessment of Reduction in Mortality and morbidity’ programme).

## 1 INTRODUCTION

Many clinical trials comprise a non‐fatal event type that can occur repeatedly, as primary or secondary endpoint. The analysis of such recurrent event data is well established and there is a plenty of statistical models for treatment effect estimation (Cook & Lawless, 2007; Kelly & Lim, 2000). However, in some situations the occurrence of a competing, terminal event must be considered, which precludes the occurrence of further recurrent events. Such kind of data particularly arise in clinical heart failure trials where treatment effects both on mortality (assessed as cardiovascular death, CV death) and morbidity (assessed as recurrent non‐fatal hospitalisations) are to be evaluated (Zannad et al., 2013; Zanolla & Zardini, 2003). One example is the CHARM‐Preserved trial (where CHARM is the ‘Candesartan in Heart failure Assessment of Reduction in Mortality and morbidity’ programme), a large multicentre trial in heart failure disease. Here treatment with the drug Candesartan did only affect the hospitalisation rate, but not mortality (Yusuf et al., 2003).

The CHARM‐Preserved trial is only one of many heart failure trials, where a composite of heart failure hospitalisations (HFHs) and CV death was defined as the primary endpoint, with a time to first event analysis as primary analysis (Anker et al., 2016). This procedure is criticised for a long time, as only a part of the data is integrated in the analysis (Anker & McMurray, 2012). In order to capture the whole disease burden, alternative statistical approaches were suggested, for example to simultaneously consider death and hospitalisations as events in Poisson or negative binomial regression (Rogers et al., 2012; Rogers, Jhund, et al., 2014; Rogers, Pocock, et al., 2014). Hence, all available data are incorporated in the treatment effect estimate. However, every approach of aggregating all the available information into a single summary measure for the treatment effect does not differentiate between the treatment effects on mortality and morbidity (Ferreira‐González et al., 2007). For this reason statistical models that allow to investigate both the effect on mortality and the effect on the hospitalisation rate, are of scientific interest.

Recently, a joint frailty regression model has been recommended to simultaneously evaluate treatment effects on HFHs and CV death whilst accounting for a potential association between the two processes (Rogers, Yaroshinsky, Pocock, Stokar, & Pogoda, 2016). Here a frailty term acts multiplicatively on both hazard rates, reflecting unexplained heterogeneity and inducing an association between the two event processes. In heart failure trials a positive association in the sense that, even conditional on covariates, a high hospitalisation risk comes along with a high mortality risk, seems plausible. Conditional on frailty, that is, on the subject's level, the joint frailty model implies a proportional hazards assumption for both risk processes (Huang & Liu, 2007; Liu, Wolfe, & Huang, 2004; Rondeau, Commenges, & Joly, 2003; Rondeau, Mathoulin‐Pelissier, Jacqmin‐Gadda, Brouste, & Soubeyran, 2007).

Even though the joint frailty model seems to reflect cardiovascular disease courses appropriately, in practice the effects on morbidity and mortality are still more often quantified by means of marginal proportional hazards models, that is, by the Cox model (Cox, 1972) for CV death and the Andersen–Gill model (Andersen & Gill, 1982) for the hospitalisation rate, respectively. Marginally (i.e. on the population's level), the proportional hazards assumption will be violated in the presence of a joint frailty term and treatment effect estimates relying on marginal proportional hazards models are expected to differ from those of conditional proportional hazards models (Aalen, Cook, & Røysland, 2015). Although results derived from joint frailty and marginal models have been contrasted for single case studies (Rogers, Jhund, et al., 2014; Rogers, Pocock, et al., 2014; Rogers, Yaroshinsky, et al., 2016), there exists no systematic investigation of the differences in effect estimates. However, this is strongly needed not only for a proper interpretation of trial results but also for a careful planning of a clinical trial as power depends on effect size. For this reason, we investigate analytically the properties of marginal model estimates in the situation where the data‐generating process actually corresponds to a joint frailty model.

In our derivations we use, that a maximum partial likelihood estimator (MPLE) in a misspecified proportional hazards model converges with sample size to a least false parameter, that is, a weighted average of the marginal hazard ratios over time (Struthers & Kalbfleisch, 1986). This result has already been used to investigate the consequences of non‐proportional event‐specific hazards in multistate models (Grambauer, Schumacher, & Beyersmann, 2010) and of covariate omission (Bretagnolle & Huber‐Carol, 1988; Schmoor & Schumacher, 1997) or functional misspecification of covariates (Gerds & Schumacher, 2001) in the Cox model. Furthermore, Henderson and Oman (1999) and Cécilia‐Joseph, Auvert, Broët, and Moreau (2015) used Struther's results to study the effects of non‐consideration of unobserved heterogeneity in univariate survival analysis. However, the theory of least false parameters has not yet been applied to investigate the consequences of an association between the risk rates for recurrent and terminal events on marginal treatment effect estimates and rejection probabilities for the recurrent event outcome. This paper fills that gap: our findings explain the relation between marginal and joint frailty estimates, which is a relevant and as yet unanswered question in heart failure trials. Thus, they can contribute to the ongoing debate on defining an appropriate statistical model for recurrent hospitalisations and CV death (Anker et al., 2016).

The paper is organised as follows. In Section 2, we will introduce to the general framework of modelling recurrent events in the presence of a terminating event. The marginal hazard ratios as a function of time are derived in Section 3. In Section 4, we analytically derive results on the asymptotic marginal treatment effect estimates. In Section 5, these findings are related to clinical trials, for example the CHARM‐Preserved trial in chronic heart failure. Simulation studies for the investigation of finite sample properties of the marginal analysis including rejection probabilities are given in Section 6. Finally, in Section 7 we conclude with a discussion where clinical trial results are interpreted in the light of our new results.

## 2 MODELS AND NOTATIONS

Let *D* be the terminal event (death) time, *M* be the number of recurrent events (hospitalisations) within [0, *D*], $T_{1}<T_{2}<\cdots <T_{M}\leq T_{0}=\infty$ the corresponding recurrent event times and *C* a censoring time. We are interested in the bivariate counting process $N\left(t\right)={\left(N_{1}\left(t\right),N_{2}\left(t\right)\right)}^{\prime }$ with elements

(1) $$N_{1}\left(t\right)=\sum_{k=0}^{M}I\left(T_{k}\leq t\right)\;\text{and}\;N_{2}\left(t\right)=I\left(D\leq t\right)$$

that are counting the events of the respective outcome over time. Let $t^{-}$ denote a time that is infinitesimally smaller than *t*. For Δ>0, the increment $\Delta N_{i}\left(t\right)=\left(N_{i}{\left(t+\Delta \right)}^{-}-N_{i}\left(t^{-}\right)\right)$ is the number of events occurring in the time interval [t,t+Δ) in the respective process (i=1,2). Let further *X* denote a *p*‐dimensional vector representing known covariates and let $H\left(t\right)=\{X,N_{1}\left(t\right),N_{2}\left(t\right),0\leq s<t\}$ represent the observed process history up to time *t*.

The hazards of the two counting process components are defined as the instantaneous rates of occurrence for a recurrent and for a terminal event, respectively, among individuals still alive and conditional on the process history:

(2) $$r_{i}\left(t|H\left(t\right)\right)=\lim_{\Delta ↘0}\frac{P\left(\Delta N_{i}\left(t\right)=1\;|\;H\left(t\right),D\geq t\right)}{\Delta }\;\left(i=1,2\right).$$

Let Y(t)=I(D≥t) be the at‐risk‐indicator. Then $Y\left(t\right)r_{i}\left(t|H\left(t\right)\right)$ (i=1,2) is the intensity of the respective counting process component.

The processes *N*_1_ and *N*_2_ are by definition dependent, as no recurrent events occur after death. But also beyond that, there might be an association between the event processes. To define that formally, consider the rate of the recurrent event process given the process history and the terminal event time D=s:

(3) $$r_{1}^{*}\left(t|H\left(t\right),D=s\right)=\lim_{\Delta ↘0}\frac{P\left(\Delta N_{1}\left(t\right)=1\;|\;H\left(t\right),D=s\right)}{\Delta }.$$

Then $r_{1}^{*}\left(t|H\left(t\right),D=s\right)=0$ for t≥s. We consider the two processes as ‘associated’, if the rate $r_{1}^{*}\left(t|H\left(t\right),D=s\right)$ does, for t<s, depend on *s*. This means that a subject's time of death is informative for its prior recurrent event rate.

The use of frailty variables, which represent risk factors that are not covered by the known covariates, has been proposed for modelling such an association of the event processes (Huang & Liu, 2007; Liu et al., 2004; Rondeau et al., 2003, 2007). We will first introduce a correlated frailty model, which is very flexible regarding the form of association. Thereafter we will consider a joint frailty model, which is more restrictive with respect to the kind of dependency between the processes.

Instead of modelling the hazards $r_{i}\left(t|H\left(t\right)\right)$ directly, frailty models are modelling conditional hazards. In general, we may think of positive random variables *Z* and *W*, without making any assumptions on their joint distribution. The frailties *Z* and *W* are assumed to be independent of the known covariates *X*. The conditional hazards are defined as follows:

(4) $$\begin{array}{cc} \lambda_{1}\left(t|H\left(t\right),Z\right) & =\lim_{\Delta ↘0}\frac{P\left(\Delta N_{1}\left(t\right)=1\;|\;Z,H\left(t\right),D\geq t\right)}{\Delta }\\ \lambda_{2}\left(t|H\left(t\right),W\right) & =\lim_{\Delta ↘0}\frac{P\left(\Delta N_{2}\left(t\right)=1\;|\;W,H\left(t\right),D\geq t\right)}{\Delta }. \end{array}$$

The conditional hazards within the correlated frailty model are modelled as

(5) $$\begin{array}{cc} \lambda_{1}\left(t|H\left(t\right),Z\right) & =\lambda_{1}\left(t|X,Z\right)=Z\lambda_{10}\left(t\right)\exp\left(\beta_{1}^{\prime }X\right)\\ \lambda_{2}\left(t|H\left(t\right),W\right) & =\lambda_{2}\left(t|X,W\right)=W\lambda_{20}\left(t\right)\exp\left(\beta_{2}^{\prime }X\right). \end{array}$$

In the correlated frailty model $\lambda_{10}\left(t\right)$, $\lambda_{20}\left(t\right)$ and β_1_, β_2_ are unspecified baseline hazard rates and regression coefficient vectors for the two event processes. The model contains a proportional hazards assumption on the subject's level (i.e. for the conditional hazards) and the censoring time *C* is assumed to be independent. The association between the two event processes is solely captured by the dependency between the two frailty variables – meaning that the process history (except for covariates) is excluded from the conditional hazards and that $N_{1}\left(t|X,Z\right)$ is a Poisson process. The processes *N*_1_ and *N*_2_ are associated on the marginal level, as shown in Appendix A.1.

Without pre‐specification of the joint distribution of the frailties *Z* and *W*, model (5) is not identifiable. For this reason, further assumptions, for example W=Z or $W=Z^{\alpha }$ with *Z* having a particular distribution were discussed (Huang & Liu, 2007; Liu et al., 2004; Rondeau et al., 2003, 2007). Conveniently, a gamma or a log‐normal distribution with mean E(Z)=1 and variance Var(Z)=θ is used for that purpose.

Below we will mainly focus on the case $W=Z^{\alpha }$. In line with other authors (see Huang & Liu, 2007; Liu et al., 2004; Rondeau et al., 2003, 2007) we will refer to that model as joint frailty model:

(6) $$\begin{array}{cc} \lambda_{1}\left(t|X,Z\right) & =Z\lambda_{10}\left(t\right)\exp\left(\beta_{1}^{\prime }X\right)\\ \lambda_{2}\left(t|X,Z\right) & =Z^{\alpha }\lambda_{20}\left(t\right)\exp\left(\beta_{2}^{\prime }X\right). \end{array}$$

Both the frailty variance θ and the α‐parameter determine the degree of association between the two event processes. The frailty variance is additionally a measure for the correlation between subsequent recurrent event times.

## 3 MARGINAL HAZARDS IN THE CORRELATED FRAILTY MODEL

As mentioned before, the hazard functions $r_{1}\left(t|H\left(t\right)\right)$ and $r_{2}\left(t|H\left(t\right)\right)$, which are defined in (2), are not modelled explicitly within the correlated frailty model. However, for a particular distribution of *Z* and *W*, they are implicitly given by

(7) $$\begin{array}{cc} r_{1}\left(t|H\left(t\right)\right) & =r_{1}\left(t|X\right)=E_{Z}\left(\lambda_{1}\left(t|X,Z\right)\right)\\ r_{2}\left(t|H\left(t\right)\right) & =r_{2}\left(t|X\right)=E_{W}\left(\lambda_{2}\left(t|X,W\right)\right). \end{array}$$

As we introduced $\lambda_{1}\left(t|X,Z\right)$ and $\lambda_{2}\left(t|X,W\right)$ as the conditional hazards, we will call $r_{1}\left(t|X\right)$ and $r_{2}\left(t|X\right)$ the marginal hazards from now on. If the two event processes are associated according to the correlated frailty model (5), the marginal hazards are in general not proportional anymore, as shown below for both endpoints. Due to selection effects, a time‐constant treatment effect on the subject's level will become time‐dependent on the population's (marginal) level. In the following, we will briefly derive the marginal hazards and their ratios as a function of time. Thereby, we will focus on the situation of a two‐arm randomised controlled trial (RCT). Hence, we consider *X* to be a binary Bin(1,p) distributed covariate indicating the treatment group. The results will afterwards be used to investigate how regression coefficient estimation is affected, if the estimation relies on (in general misspecified) marginal proportional hazards models.

For the derivation of the marginal hazards we will use the Laplace transform of the distribution of a random variable *U*, which is defined as

(8) $$L_{U}\left(s\right)=E\left(\exp(-sU)\right)=\int \exp\left(-su\right)f_{U}\left(u\right)\;du$$

with $f_{U}$ being the probability density function of *U*. The first derivative of $L_{U}$ is given by

(9) $$L_{U}^{\prime }\left(s\right)=\frac{d}{ds}\int \exp\left(-su\right)f_{U}\left(u\right)\;du=\int -u\exp\left(-su\right)f_{U}\left(u\right)\;du.$$

If the two risk processes are associated according to the correlated frailty model (5), the marginal hazards are given by

(10) $$\begin{array}{cc} r_{1}\left(t|X\right) & =\lambda_{10}\left(t\right)\exp\left(\beta_{1}X\right)\cdot E\left(Z|X,D\geq t\right) \end{array}$$

(11) $$\begin{array}{cc} r_{2}\left(t|X\right) & =\lambda_{20}\left(t\right)\exp\left(\beta_{2}X\right)\cdot E\left(W|X,D\geq t\right) \end{array}$$

with conditional expectations being dissolved as

(12) $$E\left(Z|X,D\geq t\right)=\frac{\int_{0}^{\infty }\int_{0}^{\infty }z\;\exp\left(-w\exp\left(\beta_{2}X\right)\Lambda_{20}\left(t\right)\right)f_{Z,W}\left(z,w\right)\;dw\;dz}{L_{W}\left(\exp\left(\beta_{2}X\right)\Lambda_{20}\left(t\right)\right)}$$

(13) $$E\left(W|X,D\geq t\right)=-\frac{L_{W}^{\prime }\left(\exp\left(\beta_{2}X\right)\Lambda_{20}\left(t\right)\right)}{L_{W}\left(\exp\left(\beta_{2}X\right)\Lambda_{20}\left(t\right)\right)}\;.$$

Here $\Lambda_{20}\left(t\right)=\int_{0}^{t}\lambda_{20}\left(s\right)\;ds$ denotes the cumulative baseline hazard for mortality. Details of these derivations are given in Appendix A.2. In the special case of W=Z the numerator in (12) simplifies to $-L_{W}^{\prime }\left(\exp\left(\beta_{2}X\right)\Lambda_{20}\left(t\right)\right)$ so that (12) and (13) coincide.

Using (10)–(13), the marginal hazard ratios for the two endpoints are given by

(14) $$\begin{array}{ccc} \frac{r_{1}\left(t|X=1\right)}{r_{1}\left(t|X=0\right)} & = & \exp\left(\beta_{1}\right)\cdot \frac{E(Z|X=1,D\geq t)}{E(Z|X=0,D\geq t)}\\ & = & \exp(\beta_{1})\\ & & \cdot \;\frac{L_{W}\left(\Lambda_{20}\left(t\right)\right)\cdot \int_{0}^{\infty }\int_{0}^{\infty }z\;\exp\left(-w\exp\left(\beta_{2}\right)\Lambda_{20}\left(t\right)\right)f_{Z,W}\left(z,w\right)\;dw\;dz}{L_{W}\left(\exp\left(\beta_{2}\right)\Lambda_{20}\left(t\right)\right)\cdot \int_{0}^{\infty }\int_{0}^{\infty }z\;\exp\left(-w\Lambda_{20}\left(t\right)\right)f_{Z,W}\left(z,w\right)\;dw\;dz} \end{array}$$

(15) $$\begin{array}{ccc} \frac{r_{2}\left(t|X=1\right)}{r_{2}\left(t|X=0\right)} & = & \exp\left(\beta_{2}\right)\cdot \frac{E(W|X=1,D\geq t)}{E(W|X=0,D\geq t)}\;\\ & = & \exp(\beta_{2})\\ & & \cdot \;\frac{L_{W}\left(\Lambda_{20}\left(t\right)\right)\cdot L_{W}^{\prime }\left(\exp\left(\beta_{2}\right)\Lambda_{20}\left(t\right)\right)}{L_{W}\left(\exp\left(\beta_{2}\right)\Lambda_{20}\left(t\right)\right)\cdot L_{W}^{\prime }\left(\Lambda_{20}\left(t\right)\right)}. \end{array}$$

Both marginal hazard ratios can be dissected into the product of the conditional hazard ratio and a deviation factor. Again, in the special case of W=Z, the deviation factors in the hazard ratios of both endpoints coincide. The deviation factors of both endpoints depend on the frailty distributions, on the covariate‐effect β_2_ on the terminal event rate and on the cumulative baseline terminal event rate $\Lambda_{20}\left(t\right)$. As the latter is a function of time, the marginal hazard ratios are time‐dependent in contrast to the time‐constant conditional hazard ratios,

(16) $$\begin{array}{c} \frac{\lambda_{1}\left(t|X=1,Z\right)}{\lambda_{1}\left(t|X=0,Z\right)}=\exp\left(\beta_{1}\right)\;\;\;\text{and}\;\;\;\frac{\lambda_{2}\left(t|X=1,W\right)}{\lambda_{2}\left(t|X=0,W\right)}=\exp\left(\beta_{2}\right). \end{array}$$

It is worth mentioning that the deviation between the marginal and the conditional hazard ratio over time is independent of the regression coefficient β_1_ and the cumulative baseline hazard $\Lambda_{10}\left(t\right)$ that describe the recurrent event process. This holds for both endpoints (recurrent and terminal).

## 4 ASYMPTOTICS OF MARGINAL HAZARD RATIO ESTIMATES

We will now derive asymptotic characteristics of marginal parameter estimates (log‐hazard‐ratio estimates) that erroneously rely on marginal proportional hazards models, when in fact the proportional hazards assumption holds on the conditional (subject's) level. Thus, instead of model (5), an Andersen–Gill model for the recurrent events and a Cox model for the terminal event is assumed. Both do not include the frailty variables *Z* and *W*. As before, we consider a two‐arm RCT setting with *X* being binary. Under the Andersen–Gill and Cox modelling assumptions, regression coefficients are estimated by maximum partial likelihood estimators (MPLEs) (Andersen & Gill, 1982; Cox, 1972).

Let $T_{i1}<T_{i2}<\cdots <T_{iM_{i}}\leq T_{i0}=\infty$ denote the recurrent event times, $C_{i}$ the right‐censoring time, $D_{i}$ the time of death and $X_{i}$ the binary covariate for the *i*th of *n* subjects. The MPLEs relying on the marginal models are given by

(17) $$\begin{array}{cc} {\hat{\beta }}_{1} & =\underset{\beta \in R}{\mathrm{argmax}}\;L_{1}\left(\beta \right)\;\;\;\text{with}\;\;\;L_{1}\left(\beta \right)=\prod_{i=1}^{n}\;\;\prod_{j:T_{ij}<C_{i}}\frac{\exp(\beta X_{i})}{\sum_{k\in R(T_{ij})}\exp\left(\beta X_{k}\right)} \end{array}$$

(18) $$\begin{array}{cc} {\hat{\beta }}_{2} & =\underset{\beta \in R}{\mathrm{argmax}}\;L_{2}\left(\beta \right)\;\;\;\text{with}\;\;\;L_{2}\left(\beta \right)=\prod_{i:D_{i}<C_{i}}\frac{\exp(\beta X_{i})}{\sum_{k\in R(D_{i})}\exp\left(\beta X_{k}\right)}\;. \end{array}$$

R(t) is the set of subjects being at risk at time *t*, that is, $R\left(t\right)=\{k\;|\;\left(D_{k}\wedge C_{k}\right)\geq t\}$. In the Andersen–Gill estimator ${\hat{\beta }}_{1}$, death is handled as independent censoring.

As shown by Struthers and Kalbfleisch (1986), the MPLE's ${\hat{\beta }}_{1}$ and ${\hat{\beta }}_{2}$ converge (in probability) to some least false parameters $\beta_{1}^{*}$ and $\beta_{2}^{*}$, respectively. By applying Struther and Kalbfleisch's general results to our situation, the least false parameter $\beta_{i}^{*}$ (i=1,2) can be identified as being the unique solution of the parameter integral equation $g_{i}\left(s\right)=0$ with

(19) $$\begin{array}{c} g_{i}\left(s\right)=\int_{0}^{t_{max}}\frac{{\bar{y}}^{\left(0\right)}\left(t\right){\bar{y}}^{\left(1\right)}\left(t\right)}{{\bar{y}}^{\left(0\right)}\left(t\right)+{\bar{y}}^{\left(1\right)}\left(t\right)\exp\left(s\right)}\left[r_{i}\left(t|X=1\right)-\exp\left(s\right)r_{i}\left(t|X=0\right)\right]\;dt\;. \end{array}$$

Here $t_{max}$ denotes the maximum of observation times. In addition, ${\bar{y}}^{\left(k\right)}\left(t\right)$ denotes the probability that a randomly selected subject belongs to treatment group k∈{0,1} and is still at risk at time *t*, that is,

(20) $$\begin{array}{c} {\bar{y}}^{\left(k\right)}\left(t\right)=P\left(X=k\right)S\left(t|X=k\right)P\left(C>t\right)\;. \end{array}$$

In the following, we apply (19) to investigate the asymptotic behaviour of marginal estimates for various settings. Thereby we will focus on the Andersen–Gill estimator ${\hat{\beta }}_{1}$, as the properties of the Cox estimator ${\hat{\beta }}_{2}$ in the presence of unexplained heterogeneity are already well investigated by other authors (see Cécilia‐Joseph et al., 2015; Henderson & Oman, 1999; Schmoor & Schumacher, 1997).

### 4.1 No treatment effect on mortality ($\beta_{2}=0$)

First of all, we will make some weak assumptions regarding censoring and baseline hazards: we will assume that the baseline hazards $\lambda_{10}\left(t\right)$ and $\lambda_{20}\left(t\right)$ are continuous functions which implies that the marginal hazard $r_{1}\left(t|X=k\right)$ and the marginal survival function S(t|X=k) are likewise continuous. By further assuming censoring to be continuous on $\left(0,t_{max}\right)$, we obtain ${\bar{y}}^{\left(k\right)}\left(t\right)$ to be continuous on $\left(0,t_{max}\right)$. After Cauchy's mean value theorem there exists a $t^{*}\in \left(0,t_{max}\right)$ so that

(21) $$\begin{array}{c} 0=\left[r_{1}\left(t^{*}|X=1\right)-\exp\left(\beta_{1}^{*}\right)r_{1}\left(t^{*}|X=0\right)\right]\int_{0}^{t_{max}}\frac{{\bar{y}}^{\left(0\right)}\left(t\right){\bar{y}}^{\left(1\right)}\left(t\right)}{{\bar{y}}^{\left(0\right)}\left(t\right)+{\bar{y}}^{\left(1\right)}\left(t\right)\exp\left(\beta_{1}^{*}\right)}\;dt\;. \end{array}$$

As the integral is strictly positive, we obtain

(22) $$\begin{array}{c} \beta_{1}^{*}=\log\left(\frac{r_{1}\left(t^{*}|X=1\right)}{r_{1}\left(t^{*}|X=0\right)}\right)\;. \end{array}$$

So the least false parameter $\beta_{1}^{*}$ is in the value range of the logarithmised marginal hazard ratio over the interval $\left(0,t_{max}\right)$.

If the treatment has no effect on mortality, that is, $\beta_{2}=0$, the marginal hazard ratio is constant over time and coincides with the conditional hazard ratio $\exp(\beta_{1})$ as is evident from (14). Applying (22) shows that the Andersen–Gill estimator asymptotically coincides with the conditional treatment effect for recurrent events, that is,

(23) $$\begin{array}{c} \beta_{1}^{*}\overset{\left(22\right)}{=}\log\left(\frac{r_{1}\left(t^{*}|X=1\right)}{r_{1}\left(t^{*}|X=0\right)}\right)\overset{\beta_{2}=0}{=}\beta_{1} \end{array}$$

as the latter equation is true for all *t* according to (14). This is illustrated in Figures 2, 3, 4 and in Table 3 for various settings in a joint gamma frailty model. But the result generally holds in every kind of correlated frailty model. Even though the marginal hazards $r_{1}\left(t|X=1\right)$ and $r_{1}\left(t|X=0\right)$ do not correspond to the their conditional counterparts $\lambda_{1}\left(t|X=1,Z\right)$ and $\lambda_{1}\left(t|X=0,Z\right)$, their respective ratios are equal. If treatment has no effect on mortality, the selection due to unexplained heterogeneity affects the treatment and the control group in the same way, resulting in a still proportional time course of the marginal hazards. Hence this is in fact the only situation, where the proportional hazards assumption is not violated on the marginal level. However, it should be underlined, that the processes are associated even if $\beta_{2}=0$.

### 4.2 Joint frailty model

The least false parameter $\beta_{1}^{*}$ can be derived by numerically solving $g_{1}\left(s\right)=0$ (see formula (19)) after specifying the correlated frailty model. To characterise the marginal treatment effect estimate for recurrent events more in detail, we will consider the joint frailty model (i.e. $W=Z^{\alpha }$, see formula (6)) from now on. We will show results for a gamma‐distributed frailty *Z* with mean E(Z)=1 and variance Var(Z)=θ. However, subsequent results do qualitatively not change if a log‐normal distribution is adopted (results not shown). Furthermore, we will consider a 1:1 allocation ratio and administrative censoring after $t_{max}$ time units. The subject‐specific hazards are assumed to originate from Weibull distributions. Thereby the conditional baseline hazard for the *i*th endpoint (1=recurrent, 2=terminal) is given by $\lambda_{i0}\left(t\right)=\lambda_{i}\nu_{i}t^{\nu_{i}-1}$ , with $\lambda_{i}$ being the scale parameter and $\nu_{i}$ being the shape parameter. The latter determines, if the hazard is decreasing ($\nu_{i}<1$), constant ($\nu_{i}=1$) or increasing ($\nu_{i}>1$) over time.

**Figure 1:**
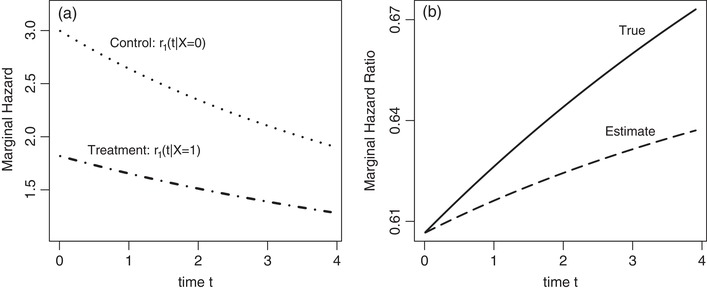
Marginal hazards and hazard ratios in a joint gamma frailty model with frailty variance θ=1, association parameter α=0.8, constant subject‐specific hazards (Weibull scale parameters $\lambda_{1}=3$ and $\lambda_{2}=0.178$, Weibull shape parameters $\nu_{1}=1$ and $\nu_{2}=1$) and treatment being protective both for hospitalisations and for mortality ($\exp\left(\beta_{1}\right)=\exp\left(-0.5\right)=0.607$, $\exp\left(\beta_{2}\right)=\exp\left(-0.3\right)=0.741$). (a) Marginal hazards for recurrent events in the treatment and control group. (b) True: marginal hazard ratio for recurrent events. Estimate: hazard ratio estimate $\exp(\beta_{1}^{*})$ resulting from a marginal Andersen–Gill analysis after administrative censoring at time point *t*

Figure 1a shows the time course of the marginal recurrent event hazards for the situation where both processes are positively associated (α=0.8), meaning that patients with a high mortality risk also have a high risk for recurrent events and vice versa. We further consider that treatment is protective with regard to both endpoints ($\beta_{1}<0$, $\beta_{2}<0$) and that the conditional baseline hazards for both endpoints are constant over time. At the time point of randomisation (t=0), treatment and control group do not differ with regard to the distribution of the frailty variables. Due to dropouts of frail patients, the marginal hazard is decreasing over time in both groups. But as selection effects differ between the groups ($\beta_{2}<0$), that decrease is non‐proportional. Hence, the marginal hazard ratio corresponds to the conditional one only at time point 0 and is afterwards increasing over time, as shown as ‘True’ in Figure 1b. It is further demonstrated that the estimated marginal hazard ratio $\exp(\beta_{1}^{*})$ at time point $t_{\max}=t$ is a weighted average of the marginal hazard ratios over the interval (0, *t*), shown as ‘Estimate’ in Figure 1b. For this reason, the marginal hazard ratio estimate depends on the length of follow‐up.

**Figure 2:**
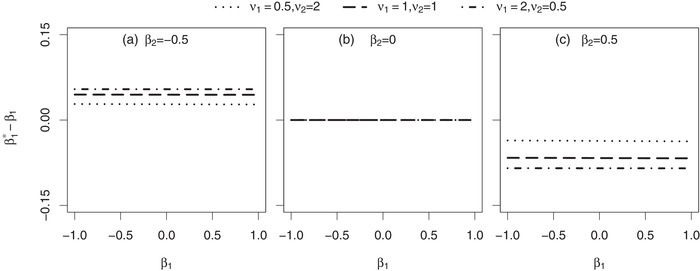
Asymptotic difference between the marginal treatment effect estimate $\beta_{1}^{*}$ and the conditional treatment effect β_1_ in a joint gamma frailty model with frailty variance θ=1 and association parameter α=0.8. *Note*: Subject‐specific hazards originate from Weibull distributions (scale parameters $\lambda_{1}=3$, λ_2_ and shape parameters ν_1_, ν_2_). The scale‐parameter λ_2_ is not shown here, but was in each scenario selected in such a way that the conditional survival probability at the end of follow‐up is S(2|X=0,Z=1)=0.7. Subjects are censored administratively after $t_{max}=2$ time units

**Figure 3:**
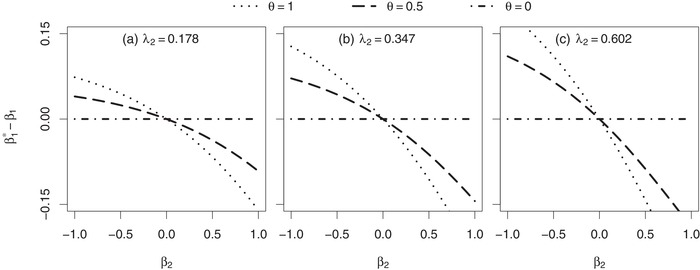
Asymptotic difference between the marginal treatment effect estimate $\beta_{1}^{*}$ and the conditional treatment effect β_1_ in a joint gamma frailty model with frailty variance θ and association parameter α=0.8. *Note*: Subject‐specific hazards are constant (Weibull scale parameters $\lambda_{1}=3$ and λ_2_, Weibull shape parameters $\nu_{1}=1$ and $\nu_{2}=1$), resulting in a conditional survival probability of S(2|X=0,Z=1)=0.7 in (a), 0.5 in (b) and 0.3 in (c) at the end of follow‐up. Subjects are censored administratively after $t_{max}=2$ time units

**Figure 4:**
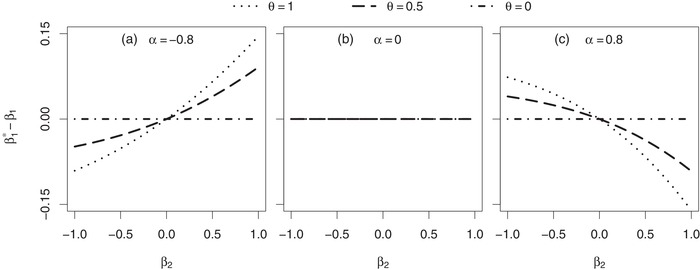
Asymptotic difference between the marginal treatment effect estimate $\beta_{1}^{*}$ and the conditional treatment effect β_1_ in a joint gamma frailty model with frailty variance θ and association parameter α. *Note*: Subject‐specific hazards are constant (Weibull scale parameters $\lambda_{1}=3$ and $\lambda_{2}=0.178$, Weibull shape parameters $\nu_{1}=1$ and $\nu_{2}=1$), resulting in a conditional survival probability of S(2|X=0,Z=1)=0.7 at the end of follow‐up. Subjects are censored administratively after $t_{max}=2$ time units

In the following we focus on the evaluation of the estimate $\beta_{1}^{*}$ after a fixed follow‐up period of $t_{max}=2$ time units. In each of Figures 2, 3, 4, the difference $\left(\beta_{1}^{*}-\beta_{1}\right)$ between the marginal treatment effect estimate (after two time units of follow‐up) and the conditional treatment effect is shown in dependence of various parameters of the joint frailty model.

First, we will focus on situations where $\beta_{1}^{*}$ and β_1_ coincide: this is the case if either $\beta_{2}=0$ (Figures 2b, 3 and 4) or α=0 (Figure 4b) or θ=0 (Figures 3 and 4). It has already been shown analytically in Subsection 4.1 that $\beta_{1}^{*}=\beta_{1}$, if treatment has no effect on mortality ($\beta_{2}=0$). If α=0, the joint frailty model reduces to a model with inter‐individual heterogeneity in recurrent events but without an association between recurrent and terminal event rates. In this situation, the Andersen–Gill estimator is consistent for the regression parameter (i.e. $\beta_{1}^{*}=\beta_{1}$), as shown by other authors (Lin, Wei, Yang, & Ying, 2000; Liu, 2014). There is likewise no association if θ=0 because then the submodel for recurrent events reduces to an Andersen–Gill model and of course β_1_ is estimated consistently by the Andersen–Gill estimator in that situation.

Next, we will take a closer look at parameters that do not affect the difference $\left(\beta_{1}^{*}-\beta_{1}\right)$: the scale parameter λ_1_ of the conditional recurrent event hazard, which mainly determines how many recurrent events are expected within the trial time, has this property. This can easily be derived analytically, as λ_1_ can be extracted as a factor in (19) and thus does not affect the solution of $g_{1}\left(s\right)=0$. The second parameter not influencing the difference $\left(\beta_{1}^{*}-\beta_{1}\right)$ is β_1_ itself, as illustrated in Figure 2 (however, an analytical proof for that result is lacking). Accordingly, results in Figures 3 and 4 do not depend on β_1_, which is for that reason no longer specified there.

Now we focus on parameters that determine the absolute difference $|\beta_{1}^{*}-\beta_{1}|$ between the marginal treatment effect estimate and the conditional treatment effect. An increase in the frailty variance θ (Figures 3 and 4) and an increase in the absolute value of the association parameter |α| (Figure 4) result in an increase of that absolute difference. In addition, increasing the mortality risk during follow‐up leads to the same result (Figure 3). As illustrated in Figure 2, the absolute difference further depends on the ratio $\nu_{1}/\nu_{2}$ between the shapes of the baseline hazards. The larger the $\nu_{1}/\nu_{2}$ ratio, the less recurrent events can occur before death and the more the marginal treatment effect estimate deviates from the conditional treatment effect.

In particular, the treatment effect on mortality β_2_ and the association parameter α determine, if the marginal treatment effect estimate $\beta_{1}^{*}$ is smaller, equal or larger than the conditional treatment effect β_1_, that is, they specify the sign of the difference $\left(\beta_{1}^{*}-\beta_{1}\right)$. This becomes apparent in Figure 4: if α>0, the difference is positive in case of $\beta_{2}<0$ and negative in case of $\beta_{2}>0$ (Figure 4c). For the special situation of α=1, an analytical proof for that finding is given in Appendix A.3. The effects turn around for α<0, that is, the difference is negative in case of $\beta_{2}<0$ and positive in case of $\beta_{2}>0$ (Figure 4a). The latter scenario is however less reasonable for heart failure trials. So let us have a look at particular situations that might be most realistic in heart failure trials: here a positive association (α>0) seems reasonable because patients with a high hospitalisation rate will probably die earlier than patients who are rarely in need for hospitalisation. In addition, most heart failure drugs either have a protective or negligible effect on mortality ($\beta_{2}\leq 0$). So in these settings, the marginal treatment effect estimate is larger than the conditional treatment effect ($\beta_{1}^{*}\geq \beta_{1}$). Hence, a conditional (joint frailty) analysis of the data will result in an estimate that favours treatment more compared to a marginal (Andersen–Gill) analysis. To illustrate that, we will consider the joint frailty setting that is shown in Figure 4c (α=0.8, θ=1) in greater detail: if $\beta_{2}=-0.5$, the difference of interest is given by $(\beta_{1}^{*}-\beta_{1})=0.045$. Hence in case of $\beta_{1}=-0.3$ the marginal estimate is $\beta_{1}^{*}=-0.3+0.045=-0.255$. As already emphasised before, the parameter β_1_ itself does not affect the deviation of $\beta_{1}^{*}$ from β_1_. Hence in case of $\beta_{1}=-0.02$, the marginal estimate is given by $\beta_{1}^{*}=-0.98+0.045=0.025$. This example shows that there may exist situations, in which even the direction does not match between the marginal estimate and the conditional treatment effect. Generally said, it may happen that $\text{sign}\left(\beta_{1}^{*}\right)\neq \text{sign}\left(\beta_{1}\right)$, corresponding to a misjudgement of the treatment effect direction for recurrent events.

## 5 APPLICATIONS

The ‘Candesartan in Heart failure Assessment of Reduction in Mortality and morbidity’ (CHARM) programme comprised three independent, randomised clinical trials that evaluated the benefit of Candesartan as a supplementary therapy in patients with chronic heart failure. The CHARM‐Preserved trial focussed on heart failure patients with preserved ejection fraction (defined as >40% left ventricular ejection fraction). A total of 3,023 patients met the inclusion criteria and were randomised to receive either Candesartan or Placebo in addition to the recommended standard therapy. The median follow‐up was 36.6 months. Regarding the primary endpoint of the trial, a composite of CV death and HFHs that was evaluated by a time to first event analysis, no treatment benefit could be shown (Rogers, Pocock, et al., 2014; Yusuf et al., 2003).

Table 1 shows results on CHARM‐Preserved that were reported in Rogers, Pocock, et al. (2014) and Yusuf et al. (2003). As apparent, CV death did not differ between the two groups. But patients in the Candesartan group had fewer HFHs compared to the Placebo group, which is reflected by a HFH hazard ratio of 0.71 in a marginal analysis (Andersen–Gill model) and of 0.69 in a conditional analysis with the joint gamma frailty model. We used our findings from Section 4 to derive the expected marginal hazard ratio estimates, thereby varying the frailty variance, and compare these with the observed estimates. For that, formula (19) is applied with parameters, which match the situation in CHARM‐Preserved (see Table 2). Thereby we assumed an association parameter of α=0.8, as published joint frailty results from other heart failure trials (CHARM‐Added, CHARM‐Alternative) suggest that heterogeneity might be greater with respect to HFHs than for mortality (Rogers et al., 2016). We can conclude that our results can well explain that there are only slight differences between marginal and conditional estimates in CHARM‐Preserved: in the presence of a negligible treatment effect on the terminal event (CV death), the hazard ratio estimates for recurrent events (HFHs) from marginal and conditional models nearly coincide (see $\beta_{2}=0$ from Subsection 4.1).

**Table 1:** Event numbers and (unadjusted) treatment effect estimates of the CHARM‐Preserved trial according to Rogers, Pocock, et al. (2014) and Yusuf et al. (2003)

			Hazard ratio (95% CI)	
	Placebo	Candesartan	Marginal	Conditional
Number of patients	1,509	1,514		
Total follow‐up years	4,374.03	4,424.62		
Number of CV deaths	170	170	0.99(0.80,1.22)	0.96(0.73,1.26)
Total number of heart failure hospitalisations	547	392	0.71(0.57,0.88)	0.69(0.55,0.85)

Marginal treatment effect estimates rely on the Cox model (CV death) or the Andersen–Gill model (heart failure hospitalisations). Conditional treatment effect estimates rely on a joint gamma frailty model.

**Table 2:** Expected marginal hazard ratio estimates in joint gamma frailty models that reflect the situation of the CHARM‐Preserved trial (using constant baseline hazards with rates that match the observed event numbers and administrative censoring after $t_{max}=3.05$ years follow‐up)

Parameters joint gamma frailty model						Expected marginal model estimates	
θ	α	$\exp(\beta_{1})$	$\exp(\beta_{2})$	$\lambda_{10}\left(t\right)$	$\lambda_{20}\left(t\right)$	$\exp(\beta_{1}^{*})$	$\exp(\beta_{2}^{*})$
0	0.8	0.69	0.96	0.125	0.039	0.6900	0.9600
1	0.8	0.69	0.96	0.125	0.039	0.6912	0.9613
2	0.8	0.69	0.96	0.125	0.039	0.6921	0.9624
3	0.8	0.69	0.96	0.125	0.039	0.6929	0.9633
4	0.8	0.69	0.96	0.125	0.039	0.6936	0.9641
5	0.8	0.69	0.96	0.125	0.039	0.6942	0.9648

As in CHARM‐Preserved, also in many other heart failure trials the randomised treatment groups differ only slightly in their risk for CV death (Zheng et al., 2018). Therefore, marginal and conditional hazard ratio estimates are supposed to coincide in these trials. HFHs and CV death are also the outcomes of interest in type 2 diabetes studies. Opposite to heart failure trials, substantial treatment effects for CV death were shown here (Schnell, Rydén, Standl, & Ceriello, on behalf of the D&CVD EASD Study Group, 2017). For example, we consider the EMPA‐REG trial (Zinman et al., 2015) that reported marginal hazard ratios of 0.65 for HFHs and 0.62 for CV death with a median observation time of 3.1 years. Note that the reported marginal HFH hazard ratio estimate is derived from a Cox analysis (time to first HFH) only and may therefore not exactly match to the (Andersen–Gill) estimate investigated in this paper. Following our findings, in this trial the conditional hazard ratio estimate for HFHs ought to be smaller than the marginal one, that is, $\exp\left(\beta_{1}\right)<\exp\left(\beta_{1}^{*}\right)=0.65$, if the rates for HFHs and CV death are positively associated (α>0, θ>0, see Subsection 4.2). However, as conditional estimates are not published, an empirical verification cannot be provided at this point.

## 6 SIMULATION STUDY

**Figure 5:**
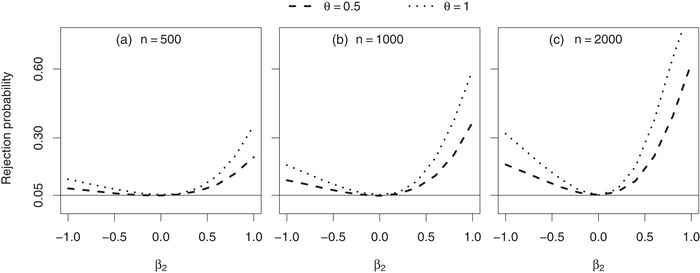
Simulation results (10,000 simulated datasets, each with *n* subjects): rejection probability of a two‐sided *Z*‐test (based on the Andersen–Gill estimate in combination with its robust standard error) for $H_{0}:\beta_{1}=0$. *Note*: Data were simulated from a joint gamma frailty model with frailty variance θ, association parameter α=0.8 and treatment effect $\beta_{1}=0$. Subject‐specific hazards are constant (Weibull scale parameters $\lambda_{1}=3$ and $\lambda_{2}=0.178$, Weibull shape parameters $\nu_{1}=1$ and $\nu_{2}=1$), resulting in a conditional survival probability of S(2|X=0,Z=1)=0.7 at the end of follow‐up. Subjects are censored administratively after $t_{max}=2$ time units

As differences between marginal and conditional hazard ratio estimates may also affect the rejection probability of the corresponding two‐sided statistical *Z*‐test (based on the Andersen–Gill estimate in combination with its robust standard error) for evaluation of the null hypothesis $H_{0}:\beta_{1}=0$, we performed simulations to investigate this effect. Data were simulated according to a joint gamma frailty model with association parameter α=0.8. We consider administrative censoring after $t_{max}=2$ time units. As before, the conditional baseline hazards for the two processes (1=recurrent, 2=terminal) originate from Weibull distributions with scale parameters λ_1_, λ_2_ and shape parameters ν_1_, ν_2_. In all scenarios, the cumulative baseline hazard for recurrent events at the end of follow‐up is $\Lambda_{10}\left(2\right)=6$, corresponding to six expected recurrent events during follow‐up if no terminal event was present. This is achieved both in a setting with a constant baseline hazard ($\lambda_{1}=3$, $\nu_{1}=1$, see Figure 5 and Table 3) and a setting with a decreasing baseline hazard ($\lambda_{1}=4.24$, $\nu_{1}=0.5$, see Table 3) for recurrent events. Furthermore, we always consider a constant baseline mortality hazard ($\lambda_{2}=0.178$, $\nu_{2}=1$), corresponding to a probability of S(2|X=0,Z=1)=0.7 to survive the follow‐up.

**Table 3:** Simulation results (10,000 simulated datasets, each with 1,000 subjects) for a marginal analysis of data from a joint gamma frailty model with association parameter α=0.8, frailty variance θ and treatment effects β_1_ (recurrent events) and β_2_ (mortality)

Simulation parameters			Recurrent events				Terminal event			
β_1_	β_2_	θ	${\hat{\beta }}_{1}-\beta_{1}$	$\beta_{1}^{*}-\beta_{1}$	$SE({\hat{\beta }}_{1})$	rp	${\hat{\beta }}_{2}-\beta_{2}$	$\beta_{2}^{*}-\beta_{2}$	$SE({\hat{\beta }}_{2})$	rp
**(a)** $\lambda_{1}=3$ and $\nu_{1}=1$										
0.0	−0.5	1.0	0.045	0.045	0.070	0.100	0.037	0.036	0.139	0.912
0.0	−0.5	2.0	0.076	0.077	0.094	0.130	0.063	0.063	0.147	0.850
0.0	0.0	1.0	0.000	0.000	0.071	0.052	0.002	0.000	0.125	0.053
0.0	0.0	2.0	−0.001	0.000	0.094	0.049	0.001	0.000	0.132	0.051
0.0	0.5	1.0	−0.066	−0.067	0.072	0.155	−0.053	−0.055	0.115	0.974
0.0	0.5	2.0	−0.111	−0.111	0.094	0.224	−0.089	−0.092	0.122	0.927
−0.5	−0.5	1.0	0.043	0.045	0.072	1.000	0.034	0.036	0.140	0.916
−0.5	−0.5	2.0	0.077	0.077	0.095	0.994	0.063	0.063	0.147	0.850
−0.5	0.0	1.0	−0.002	0.000	0.073	1.000	−0.001	0.000	0.125	0.048
−0.5	0.0	2.0	0.000	0.000	0.096	0.999	0.001	0.000	0.132	0.051
−0.5	0.5	1.0	−0.068	−0.067	0.074	1.000	−0.054	−0.055	0.115	0.976
−0.5	0.5	2.0	−0.111	−0.110	0.096	1.000	−0.091	−0.092	0.122	0.920
**(b)** $\lambda_{1}=4.24$ and $\nu_{1}=0.5$										
0.0	−0.5	1.0	0.029	0.029	0.069	0.073	0.037	0.036	0.139	0.912
0.0	−0.5	2.0	0.049	0.050	0.092	0.086	0.063	0.063	0.147	0.850
0.0	0.0	1.0	0.001	0.000	0.069	0.052	0.002	0.000	0.125	0.053
0.0	0.0	2.0	−0.001	0.000	0.091	0.048	0.001	0.000	0.132	0.051
0.0	0.5	1.0	−0.041	−0.043	0.069	0.097	−0.053	−0.055	0.115	0.974
0.0	0.5	2.0	−0.070	−0.070	0.091	0.121	−0.089	−0.092	0.122	0.927
−0.5	−0.5	1.0	0.028	0.029	0.070	1.000	0.034	0.036	0.140	0.916
−0.5	−0.5	2.0	0.049	0.050	0.093	0.998	0.063	0.063	0.147	0.850
−0.5	0.0	1.0	−0.001	0.000	0.071	1.000	−0.001	0.000	0.125	0.048
−0.5	0.0	2.0	0.000	0.000	0.093	1.000	0.001	0.000	0.132	0.051
−0.5	0.5	1.0	−0.043	−0.043	0.071	1.000	−0.054	−0.055	0.115	0.976
−0.5	0.5	2.0	−0.069	−0.070	0.093	1.000	−0.091	−0.092	0.122	0.920

Subject‐specific hazards originate from Weibull distributions with scale parameters λ_1_, λ_2_ and shape parameters ν_1_, ν_2_. We consider constant subject‐specific mortality hazards ($\lambda_{2}=0.178$, $\nu_{2}=1$) in combination both with **(a)** constant ($\lambda_{1}=3$, $\nu_{1}=1$) and **(b)** decreasing ($\lambda_{1}=4.24$, $\nu_{1}=0.5$) subject‐specific recurrent event hazards. Subjects are censored administratively after $t_{max}=2$ time units. The table shows the estimates (${\hat{\beta }}_{i}$), the robust standard errors ($SE({\hat{\beta }}_{i})$) and rejection probabilities (rp) resulting from the simulation of a marginal model analysis. In addition, the asymptotically valid least false parameters ($\beta_{i}^{*}$, numerical calculation) are shown.

In all scenarios shown in Figure 5 and in half of the scenarios shown in Table 3, no treatment effect on the recurrent event rate is present (i.e. $\beta_{1}=0$). Here the rejection probability of the test corresponds to its type I error probability. The test is exact if $\beta_{2}=0$ and anticonservative if $\beta_{2}\neq 0$. In the latter case, the rejection probability under *H*_0_ increases with sample size (Figure 5) because the Andersen–Gill estimator ${\hat{\beta }}_{1}$ converges in probability to $\beta_{1}^{*}\neq \beta_{1}=0$, while its robust standard error $SE({\hat{\beta }}_{1})$ converges in probability to 0. Besides β_2_, the rejection probability under *H*_0_ is of course also affected by all the additional joint frailty parameters that determine how much $\beta_{1}^{*}$ deviates from $\beta_{1}=0$ (see Subsection 4.2). For example, it increases by increasing the frailty variance θ (Figure 5, Table 3) and by switching from a constant to a decreasing baseline hazard for recurrent events (Table 3). Table 3 further shows scenarios with an existing treatment effect on the recurrent event rate ($\beta_{1}=-0.5$). Here the rejection probability of the test corresponds to its power. In these selected scenarios, the null hypothesis is rejected almost for certain. The reason for this is that both baseline recurrent event rate and treatment effect were chosen to be relatively large. However, power would be rather poor in scenarios where $\beta_{1}^{*}=0$ but $\beta_{1}\neq 0$.

Furthermore, the simulation results of Table 3 confirm the effects that were already observed in the numerical findings shown in Figures 2–4: the difference $\left({\hat{\beta }}_{1}-\beta_{1}\right)$ is zero in case of $\beta_{2}=0$, while being positive for $\beta_{2}<0$ and negative for $\beta_{2}>0$. These directions of deviation are caused by the positive association of α=0.8 (compare with Figure 4c). In addition, the magnitude of the difference $\left({\hat{\beta }}_{1}-\beta_{1}\right)$ is not affected by the treatment effect β_1_ itself (compare with Figure 2), but is increasing with the frailty variance θ (compare with Figures 3 and 4). The difference is smaller in the scenario with a decreasing subject‐specific recurrent event hazard because here a subject has on average more recurrent events before death may terminate its follow‐up compared to the scenario with a constant recurrent event hazard (compare with Figure 2a).

Although this paper is primarily dealing with the recurrent event endpoint, Table 3 also shows estimates and rejection probabilities of a marginal analysis for the terminal event (using the Cox model). Of course, the difference $\left({\hat{\beta }}_{2}-\beta_{2}\right)$ is not affected by parameters that solely refer to recurrent events, for example the treatment effect β_1_ and the shape of the hazard for recurrent events. But the difference is increasing with the frailty variance θ and strongly depends on β_2_: although ${\hat{\beta }}_{2}=\beta_{2}$ in case of $\beta_{2}=0$, ${\hat{\beta }}_{2}$ is always shifted towards zero (relative to β_2_) in case of $\beta_{2}\neq 0$. Importantly, the Cox analysis is keeping the type I error despite unobserved heterogeneity. These results are in line with those of other authors who already investigated the behaviour of Cox model estimation in the presence of unobserved heterogeneity (see Cécilia‐Joseph et al., 2015; Henderson & Oman, 1999; Schmoor & Schumacher, 1997).

In each scenario shown in Table 3, we see slight differences between ${\hat{\beta }}_{1}$ (or ${\hat{\beta }}_{2}$), that is, the result obtained by simulation, and $\beta_{1}^{*}$ (or $\beta_{2}^{*}$), that is, the result obtained by numerically solving $g_{1}\left(s\right)=0$ (or $g_{2}\left(s\right)=0$). These differences may be due to asymptotics and/or slight numerical imprecision.

## 7 DISCUSSION

In the present paper, we derive the properties of marginal hazard ratio estimates for a recurrent event in the presence of an associated terminal event. Our findings are answering the up to now unresolved question if, why and how terminal events will affect marginal hazard ratio estimates for recurrent events. These results will contribute to a proper interpretation of marginal hazard ratios that are commonly presented from clinical trials. An important field of applications are clinical studies in heart failure, which have motivated our research: although the rate of hospitalisations due to heart failure disease will most probably be associated with the risk of CV death, the treatment effect on the hospitalisation rate is often quantified by the Andersen–Gill model – an approach that is assuming proportional hazards on the marginal level.

We have derived the least false parameter $\beta_{1}^{*}$ that is consistently estimated by the Andersen–Gill estimator in a correlated frailty model by applying theory on misspecified proportional hazards models (Struthers & Kalbfleisch, 1986). Within the correlated frailty model, an association between the recurrent and the terminal event process is induced by correlated random effects *Z* and *W*. It implies the assumption of proportional hazards with treatment effects β_1_ (recurrent events) and β_2_ (terminal event) on the subject's level. Moreover, it assumes the subject‐specific counting process for the recurrent events to be of Poisson type. On the marginal level, the proportional hazards assumption does in general not hold anymore, leading to a misspecification of the Andersen–Gill model. We have shown that the least false parameter is implicitly defined by the true underlying data‐generating process, that is, it depends on parameters of the correlated frailty model (treatment effects, baseline hazards, frailty distributions). In addition, the asymptotic estimate strongly depends on the censoring distribution including especially the length of follow‐up. Using these derivations we proved that the Andersen–Gill estimator asymptotically coincides with the conditional (i.e. subject‐specific) treatment effect, if the treatment does not affect mortality. Further results were obtained numerically for the case of a joint frailty model, where the frailties affecting the two processes have a deterministic dependency: here we have shown that the sign of the difference $\left(\beta_{1}^{*}-\beta_{1}\right)$ is, amongst others, determined by the treatment effect on mortality β_2_. Moreover, our results suggest that the size of the difference $\left(\beta_{1}^{*}-\beta_{1}\right)$ is not affected by the treatment effect β_1_ itself. In particular, a misjudgement of the treatment effect direction on recurrent events is possible, that is, $\text{sign}\left(\beta_{1}^{*}\right)\neq \text{sign}\left(\beta_{1}\right)$. In this paper, we focussed on the gamma frailty as it is most widely applied (Wienke, 2010). Equal conclusions are obtained when a log‐normal frailty is applied. To investigate other frailty distributions, further numerical investigations or simulation studies could be performed.

Some authors use the term ‘bias’ for describing deviations of marginal model estimates from conditional ones. As both approaches have different purposes depending on the aim of the study, differences between marginal and conditional model estimates are not a matter of bias. Marginal estimates provide an information about the treatment effect on the population's level, whereas conditional models, which account for the association by using frailty terms, are intended for the assessment of a treatment effect on the subject's level.

As pointed out by Bretagnolle and Huber‐Carol (1988), Schmoor and Schumacher (1997) and Aalen et al. (2015), omission of relevant covariates in a usual Cox analysis for a survival outcome leads to non‐causal treatment effect estimates despite randomisation. In the present paper, we transfer these concepts to the analysis of recurrent events and show that the same issue arises in the assessment of a treatment effect on recurrent events if an associated terminal event is present. If the treatment affects mortality, a constant conditional hazard ratio (i.e. on the subject's level) translates to a time‐dependent marginal hazard ratio (i.e. on the population's level), resulting in a time‐dependent marginal hazard ratio estimate $\exp(\beta_{1}^{*})$. However, if treatment does not affect mortality, unobserved risk factors that affect both endpoints, remain balanced over time between the survivors in the treatment and the control group. Hence, the marginal treatment effect estimate coincides with the conditional treatment effect despite an association between the processes.

Recently, Rogers et al. (2016) recommended to evaluate future heart failure trials by using a joint frailty model. The authors performed simulations to study the behaviour of different marginal treatment effect estimates for the recurrent event endpoint, when informative dropouts according to a joint frailty model occur. Our findings extend these simulation results by providing numerical solutions for the Andersen–Gill estimates. Our analytical results are supported by clinical data of the CHARM‐Preserved heart failure trial. Here the Andersen–Gill estimate does only slightly differ from the joint frailty estimate for the recurrent events because mortality seems to be unaffected by the Candesartan treatment (Rogers, Pocock, et al., 2014; Yusuf et al., 2003). Nowadays, lots of heart failure drug trials fail to show a treatment effect on mortality (Zheng et al., 2018), suggesting that marginal and conditional treatment effect estimates for the hospitalisation rate would coincide – provided the assumptions of a joint frailty model hold. Our results can also be applied for diabetes trials where cardiovascular outcomes such as HFHs and CV death are also of interest (Schnell et al., 2017). Furthermore, the findings presented here are relevant for the analysis of safety data in randomised clinical trials, where the treatment effect on the rate of recurrent, adverse events is of interest (Allignol, Beyersmann, & Schmoor, 2016; Hengelbrock, Gillhaus, Kloss, & Leverkus, 2016; Schmoor, Bender, Beyersmann, Kieser, & Schumacher, 2016). In that context, treatment discontinuation (e.g. due to death) represents the terminal event that is probably associated with the risk of adverse events.

In a recent article, Putter and van Houwelingen critically discussed the use of frailties in multistate models to account for possible associations between transition times. They have pointed out that deviations from the modelling assumptions can be absorbed by the frailty term in terms of estimating an increased frailty variance (Putter & van Houwelingen, 2015). Consequently, it can be difficult to disentangle (true) heterogeneity and violations of the modelling assumptions when applying the joint frailty model. Besides assuming proportional hazards on the subject's level, a further crucial assumption of this model is that the subject‐specific hazards for both endpoints only depend on time, but not on the history of recurrent events. This is questionable and the model could be improved by allowing for event dependency on the subject's level. However, such complex models will result in identifiability problems, as it is difficult, if not impossible, to distinguish between event dependency and unexplained heterogeneity in the data.

As aforementioned, several potential pitfalls have to be considered when treatment effects are assessed by more and more complicated models. Therefore, simpler marginal models may be preferred and we need to be able to interpret their estimates, if the underlying data‐generating processes are rather complex. We contribute to the interpretation of the Andersen–Gill estimate for the treatment effect on the recurrent event rate, if informative dropouts due to an associated terminal event are present. Our results complement previous articles reporting on treatment effect estimation in a misspecified Andersen–Gill model: Lin et al. (2000) and Liu (2014) showed that marginal and conditional treatment effects coincide, if unexplained heterogeneity, but no associated terminal event is present. Cheung, Xu, Tan, Cutts, and Milligan (2010) analysed asymptotic properties of the Andersen–Gill estimator in a model with unexplained heterogeneity and event dependency, again without considering an associated terminal event. To complete the picture, a straightforward next step would be to characterise the estimator's properties if the data‐generating process contains both informative dropouts and event dependency, probably reflecting the most realistic scenario in heart failure trials.

## CONFLICT OF INTEREST

The authors declare that there is no conflict of interest.

## Supporting information

Supplementary Information

## APPENDIX 1

### A.1 Associated processes in the correlated frailty model

$$\begin{array}{cc} r_{1}^{*}\left(t|H\left(t\right),D=s\right) & =r_{1}^{*}\left(t|X,D=s\right)\\ & =\lim_{\Delta ↘0}\frac{\int_{0}^{\infty }P\left(\Delta N_{1}\left(t\right)=1|X,Z=z,D=s\right)\cdot f_{Z|X,D=s}\left(z\right)\;dz}{\Delta }\\ & =\left\{\begin{array}{c} 0,\;\;t\geq s\\ \int_{0}^{\infty }\lambda_{10}\left(t\right)\exp\left(\beta_{1}^{\prime }X\right)\cdot z\cdot f_{Z|X,D=s}\left(z\right)\;dz,\;\;t<s \end{array}\right.\\ & =\left\{\begin{array}{c} 0,\;\;t\geq s\\ \lambda_{10}\left(t\right)\exp\left(\beta_{1}^{\prime }X\right)E\left(Z|X,D=s\right),\;\;t<s. \end{array}\right. \end{array}$$

As *Z* and *D* are dependent (via *W*), the conditional expectation E(Z|X,D=s) depends on *s*. Consequently the rate $r_{1}^{*}\left(t|H\left(t\right),D=s\right)$ does, for t<s, depend on *s*. This is considered as an association of the processes throughout the paper.

### A.2 Marginal hazards in the correlated frailty model: Derivations

Derivation of marginal hazards in the correlated frailty model (for binary *X*) as given in (10) and (11):

$$\begin{array}{cc} r_{1}\left(t|X\right) & =E_{Z}\left(\lambda_{1}\left(t|X,Z\right)\right)\\ & =\lim_{\Delta ↘0}\frac{\int_{0}^{\infty }P\left(\Delta N_{1}\left(t\right)=1|X,Z=z,D\geq t\right)\cdot f_{Z|X,D\geq t}\left(z\right)\;dz}{\Delta }\\ & =\int_{0}^{\infty }\lambda_{10}\left(t\right)\exp\left(\beta_{1}X\right)\cdot z\cdot f_{Z|X,D\geq t}\left(z\right)\;dz\\ & =\lambda_{10}\left(t\right)\exp\left(\beta_{1}X\right)\cdot E\left(Z|X,D\geq t\right)\\ r_{2}\left(t|X\right) & =E_{W}\left(\lambda_{2}\left(t|X,W\right)\right)\\ & =\lim_{\Delta ↘0}\frac{\int_{0}^{\infty }P\left(\Delta N_{2}\left(t\right)=1|X,W=w,D\geq t\right)\cdot f_{W|X,D\geq t}\left(w\right)\;dw}{\Delta }\\ & =\int_{0}^{\infty }\lambda_{20}\left(t\right)\exp\left(\beta_{2}X\right)\cdot w\cdot f_{W|X,D\geq t}\left(w\right)\;dw\\ & =\lambda_{20}\left(t\right)\exp\left(\beta_{2}X\right)\cdot E\left(W|X,D\geq t\right). \end{array}$$

Let S(t|X) and S(t|X,W=w) denote the marginal and the conditional survival function, respectively. Let further $\Lambda_{20}\left(t\right)=\int_{0}^{t}\lambda_{20}\left(s\right)\;ds$ denote the cumulative baseline hazard for mortality. Using these notations, we can now derive the conditional expectation of *Z* appearing in the marginal hazard formula for the recurrent events (10):

$$\begin{array}{cc} E(Z|X,D\geq t) & =\int_{0}^{\infty }zf_{Z|X,D\geq t}\left(z\right)\;dz\\ & =\frac{1}{S(t|X)}\int_{0}^{\infty }\int_{0}^{\infty }\int_{t}^{\infty }zf_{Z,W,D|X}\left(z,w,s\right)\;ds\;dw\;dz\\ & =\frac{1}{E_{W}\left(S(t|X,W)\right)}\int_{0}^{\infty }\int_{0}^{\infty }\int_{t}^{\infty }zf_{D|X,Z=z,W=w}\left(s\right)f_{Z,W}\left(z,w\right)\;ds\;dw\;dz\\ & =\frac{1}{\int_{0}^{\infty }S\left(t|X,W=w\right)f_{W}\left(w\right)\;dw}\int_{0}^{\infty }\int_{0}^{\infty }zS\left(t|X,W=w\right)f_{Z,W}\left(z,w\right)\;dw\;dz\\ & =\frac{\int_{0}^{\infty }\int_{0}^{\infty }z\;\exp\left(-w\exp\left(\beta_{2}X\right)\Lambda_{20}\left(t\right)\right)f_{Z,W}\left(z,w\right)\;dw\;dz}{\int_{0}^{\infty }\exp\left(-w\exp\left(\beta_{2}^{\prime }X\right)\Lambda_{20}\left(t\right)\right)f_{W}\left(w\right)\;dw}\\ & =\frac{\int_{0}^{\infty }\int_{0}^{\infty }z\;\exp\left(-w\exp\left(\beta_{2}X\right)\Lambda_{20}\left(t\right)\right)f_{Z,W}\left(z,w\right)\;dw\;dz}{L_{W}\left(\exp\left(\beta_{2}X\right)\Lambda_{20}\left(t\right)\right)}. \end{array}$$

Here we used that the marginal survival function can be expressed in terms of the Laplace transform, that is $S\left(t|X\right)=L_{W}\left(\exp\left(\beta_{2}X\right)\Lambda_{20}\left(t\right)\right)$. Analogously, we can derive the conditional expectation of *W* appearing in the marginal hazard formula for the terminal event (11):

$$\begin{array}{cc} E(W|X,D\geq t) & =\int_{0}^{\infty }wf_{W|X,D\geq t}\left(w\right)\;dw\\ & =\frac{1}{S(t|X)}\int_{0}^{\infty }\int_{t}^{\infty }wf_{W,D|X}\left(w,s\right)\;ds\;dw\\ & =\frac{1}{L_{W}\left(\exp\left(\beta_{2}X\right)\Lambda_{20}\left(t\right)\right)}\int_{0}^{\infty }\int_{t}^{\infty }wf_{D|X,W=w}\left(s\right)f_{W}\left(w\right)\;ds\;dw\\ & =\frac{1}{L_{W}\left(\exp\left(\beta_{2}X\right)\Lambda_{20}\left(t\right)\right)}\int_{0}^{\infty }wS\left(t|X,W=w\right)f_{W}\left(w\right)\;dw\\ & =\frac{\int_{0}^{\infty }w\;\exp\left(-w\exp\left(\beta_{2}X\right)\Lambda_{20}\left(t\right)\right)f_{W}\left(w\right)\;dw}{L_{W}\left(\exp\left(\beta_{2}X\right)\Lambda_{20}\left(t\right)\right)}\\ & =-\frac{L_{W}^{\prime }\left(\exp\left(\beta_{2}X\right)\Lambda_{20}\left(t\right)\right)}{L_{W}\left(\exp\left(\beta_{2}X\right)\Lambda_{20}\left(t\right)\right)}. \end{array}$$

### A.3 Direction of deviation for the marginal treatment effect estimate in a joint gamma frailty model with α=1

Here we provide a proof regarding the sign of the difference $\left(\beta_{1}^{*}-\beta_{1}\right)$ for the special situation of a joint gamma frailty model with association parameter α=1. As shown by other authors (see, e.g. Wienke, 2010), the Laplace transform of a gamma distribution (with mean 1 and variance θ) and its derivative are given by

$$\begin{array}{c} L_{Z}\left(s\right)={\left(1+\theta s\right)}^{-\frac{1}{\theta }}\;\;\;\text{and}\;\;\;L_{Z}^{\prime }\left(s\right)=-{\left(1+\theta s\right)}^{-\frac{1}{\theta }-1}, \end{array}$$

respectively. Using (14) for the special case of W=Z, the marginal hazard ratio for the recurrent event endpoint simplifies to

$$\begin{array}{c} \frac{r_{1}\left(t|X=1\right)}{r_{1}\left(t|X=0\right)}=\exp\left(\beta_{1}\right)\frac{1+\theta \Lambda_{20}\left(t\right)}{1+\theta \exp\left(\beta_{2}\right)\Lambda_{20}\left(t\right)}\;\left\{\begin{array}{cc} \leq \exp(\beta_{1})\;\forall t\;\; & \Leftrightarrow \;\;\beta_{2}>0\\ =\exp(\beta_{1})\;\forall t\;\; & \Leftrightarrow \;\;\beta_{2}=0\;.\\ \geq \exp(\beta_{1})\;\forall t\;\; & \Leftrightarrow \;\;\beta_{2}<0 \end{array}\right. \end{array}$$

The sign of the treatment effect on mortality determines, in which direction the marginal hazard ratio deviates from the conditional one for every time‐point during follow‐up. Applying (22) results in

$$\begin{array}{cc} \beta_{1}^{*}\leq \beta_{1}\;\; & \Leftrightarrow \;\;\beta_{2}>0\\ \beta_{1}^{*}=\beta_{1}\;\; & \Leftrightarrow \;\;\beta_{2}=0\\ \beta_{1}^{*}\geq \beta_{1}\;\; & \Leftrightarrow \;\;\beta_{2}<0. \end{array}$$

## References

[bimj2016-bib-0001] Aalen, O. O. , Cook, R. J. , & Røysland, K. (2015). Does Cox analysis of a randomized survival study yield a causal treatment effect? Lifetime Data Analysis, 21(4), 579–593. 26100005 10.1007/s10985-015-9335-y

[bimj2016-bib-0002] Allignol, A. , Beyersmann, J. , & Schmoor, C. (2016). Statistical issues in the analysis of adverse events in time‐to‐event data. Pharmaceutical Statistics, 15(4), 297–305. 26929180 10.1002/pst.1739

[bimj2016-bib-0003] Andersen, P. K. , & Gill, R. (1982). Cox's regression model for counting processes: A large sample study. Annals of Statistics, 10(4), 1100–1120.

[bimj2016-bib-0004] Anker, S. D. , & McMurray, J. J. (2012). Time to move on from ‘time‐to‐first’: Should all events be included in the analysis of clinical trials? European Heart Journal, 33(22), 2764–2765. 22927554 10.1093/eurheartj/ehs277

[bimj2016-bib-0005] Anker, S. D. , Schroeder, S. , Atar, D. , Bax, J. J. , Ceconi, C. , Cowie, M. R. , … Ruschitzka, F. (2016). Traditional and new composite endpoints in heart failure clinical trials: Facilitating comprehensive efficacy assessments and improving trial efficiency. European Journal of Heart Failure, 18(5), 482–489. 27071916 10.1002/ejhf.516

[bimj2016-bib-0006] Bretagnolle, J. , & Huber‐Carol, C. (1988). Effects of omitting covariates in Cox's model for survival data. Scandinavian Journal of Statistics, 15(2), 125–138.

[bimj2016-bib-0007] Cécilia‐Joseph, E. , Auvert, B. , Broët, P. , & Moreau, T. (2015). Influence of trial duration on the bias of the estimated treatment effect in clinical trials when individual heterogeneity is ignored. Biometrical Journal, 57(3), 371–383. 25597640 10.1002/bimj.201400046

[bimj2016-bib-0008] Cheung, Y. B. , Xu, Y. , Tan, S. H. , Cutts, F. , & Milligan, P. (2010). Estimation of intervention effects using first or multiple episodes in clinical trials: The Andersen–Gill model re‐examined. Statistics in Medicine, 29(3), 328–336. 19941319 10.1002/sim.3783

[bimj2016-bib-0009] Cook, R. J. , & Lawless, J. F. (2007). The statistical analysis of recurrent events. New York: Springer Science & Business Media.

[bimj2016-bib-0010] Cox, D. R. (1972). Regression models and life‐tables. Journal of the Royal Statistical Society. Series B (Methodological), 34(2), 187–220.

[bimj2016-bib-0011] Ferreira‐González, I. , Busse, J. W. , Heels‐Ansdell, D. , Montori, V. M. , Akl, E. A. , Bryant, D. M. , … Guyatt, G. H. (2007). Problems with use of composite end points in cardiovascular trials: Systematic review of randomised controlled trials. British Medical Journal, 334(7597), 786. 17403713 10.1136/bmj.39136.682083.AE PMC1852019

[bimj2016-bib-0012] Gerds, T. A. , & Schumacher, M. (2001). On functional misspecification of covariates in the Cox regression model. Biometrika, 88(2), 572–580.

[bimj2016-bib-0013] Grambauer, N. , Schumacher, M. , & Beyersmann, J. (2010). Proportional subdistribution hazards modeling offers a summary analysis, even if misspecified. Statistics in Medicine, 29(7–8), 875–884. 20213713 10.1002/sim.3786

[bimj2016-bib-0014] Henderson, R. , & Oman, P. (1999). Effect of frailty on marginal regression estimates in survival analysis. Journal of the Royal Statistical Society: Series B (Statistical Methodology), 61(2), 367–379.

[bimj2016-bib-0015] Hengelbrock, J. , Gillhaus, J. , Kloss, S. , & Leverkus, F. (2016). Safety data from randomized controlled trials: Applying models for recurrent events. Pharmaceutical Statistics, 15(4), 315–323. 27291933 10.1002/pst.1757

[bimj2016-bib-0016] Huang, X. , & Liu, L. (2007). A joint frailty model for survival and gap times between recurrent events. Biometrics, 63(2), 389–397. 17688491 10.1111/j.1541-0420.2006.00719.x

[bimj2016-bib-0017] Kelly, P. J. , & Lim, L. L. (2000). Survival analysis for recurrent event data: An application to childhood infectious diseases. Statistics in Medicine, 19(1), 13–33. 10623910 10.1002/(sici)1097-0258(20000115)19:1<13::aid-sim279>3.0.co;2-5

[bimj2016-bib-0018] Lin, D. Y. , Wei, L. J. , Yang, I. , & Ying, Z . (2000). Semiparametric regression for the mean and rate functions of recurrent events. Journal of the Royal Statistical Society: Series B (Statistical Methodology), 62, 711–730.

[bimj2016-bib-0019] Liu, X. (2014). Survival models on unobserved heterogeneity and their applications in analyzing large‐scale survey data. Journal of Biometrics and Biostatistics, 5(2), 157–162. 10.4172/2155-6180.1000191 PMC4267525 25525559

[bimj2016-bib-0020] Liu, L. , Wolfe, R. A. , & Huang, X. (2004). Shared frailty models for recurrent events and a terminal event. Biometrics, 60(3), 747–756. 15339298 10.1111/j.0006-341X.2004.00225.x

[bimj2016-bib-0021] Putter, H. , & van Houwelingen, H. C. (2015). Frailties in multi‐state models: Are they identifiable? Do we need them? Statistical Methods in Medical Research, 24(6), 675–692. 22116343 10.1177/0962280211424665

[bimj2016-bib-0022] Rogers, J. K. , Jhund, P. S. , Perez, A. C. , Böhm, M. , Cleland, J. G. , Gullestad, L. , … Pocock, S. J. (2014). Effect of rosuvastatin on repeat heart failure hospitalizations: The CORONA Trial (Controlled Rosuvastatin Multinational Trial in Heart Failure). JACC: Heart Failure, 2(3), 289–297. 24952697 10.1016/j.jchf.2013.12.007

[bimj2016-bib-0023] Rogers, J. K. , McMurray, J. J. , Pocock, S. J. , Zannad, F. , Krum, H. , van Veldhuisen, D. J. , … Pitt, B. (2012). Eplerenone in patients with systolic heart failure and mild symptoms: Analysis of repeat hospitalizations. Circulation, 126(19), 2317–2323. 23042980 10.1161/CIRCULATIONAHA.112.110536

[bimj2016-bib-0024] Rogers, J. K. , Pocock, S. J. , McMurray, J. J. , Granger, C. B. , Michelson, E. L. , Östergren, J. , … Yusuf, S. (2014). Analysing recurrent hospitalizations in heart failure: A review of statistical methodology, with application to CHARM‐Preserved. European Journal of Heart Failure, 16(1), 33–40. 24453096 10.1002/ejhf.29 PMC4822681

[bimj2016-bib-0025] Rogers, J. K. , Yaroshinsky, A. , Pocock, S. J. , Stokar, D. , & Pogoda, J. (2016). Analysis of recurrent events with an associated informative dropout time: Application of the joint frailty model. Statistics in Medicine, 35(13), 2195–2205. 26751714 10.1002/sim.6853 PMC5019155

[bimj2016-bib-0026] Rondeau, V. , Commenges, D. , & Joly, P. (2003). Maximum penalized likelihood estimation in a gamma‐frailty model. Lifetime Data Analysis, 9(2), 139–153. 12735493 10.1023/a:1022978802021 PMC1961627

[bimj2016-bib-0027] Rondeau, V. , Mathoulin‐Pelissier, S. , Jacqmin‐Gadda, H. , Brouste, V. , & Soubeyran, P. (2007). Joint frailty models for recurring events and death using maximum penalized likelihood estimation: Application on cancer events. Biostatistics, 8(4), 708–721. 17267392 10.1093/biostatistics/kxl043

[bimj2016-bib-0028] Schmoor, C. , Bender, R. , Beyersmann, J. , Kieser, M. , & Schumacher, M. (2016). Adverse event development in clinical oncology trials. Lancet Oncology, 17(7), e263–e264. 10.1016/S1470-2045(16)30223-6 27396638

[bimj2016-bib-0029] Schmoor, C. , & Schumacher, M. (1997). Effects of covariate omission and categorization when analysing randomized trials with the Cox model. Statistics in Medicine, 16(1–3), 225–237. 9004394 10.1002/(sici)1097-0258(19970215)16:3<225::aid-sim482>3.0.co;2-c

[bimj2016-bib-0030] Schnell, O. , Rydén, L. , Standl, E. , Ceriello, A. , on behalf of the D&CVD EASD Study Group (2017). Updates on cardiovascular outcome trials in diabetes. Cardiovascular Diabetology, 16(1), 128. 29020969 10.1186/s12933-017-0610-y PMC5637292

[bimj2016-bib-0031] Struthers, C. A. , & Kalbfleisch, J. D. (1986). Misspecified proportional hazard models. Biometrika, 73(2), 363–369.

[bimj2016-bib-0032] Wienke, A. (2010). Frailty models in survival analysis. Boca Raton, FL: Chapman and Hall/CRC Biostatistics Series.

[bimj2016-bib-0033] Yusuf, S. , Pfeffer, M. A. , Swedberg, K. , Granger, C. B. , Held, P. , McMurray, J. J. V. , … Östergren, J. (2003). Effects of candesartan in patients with chronic heart failure and preserved left‐ventricular ejection fraction: The CHARM‐Preserved Trial. Lancet, 362(9386), 777–781. 13678871 10.1016/S0140-6736(03)14285-7

[bimj2016-bib-0034] Zannad, F. , Stein, K. , Garcia, A. A. , Anker, S. D. , Armstrong, P. W. , Calvo, G. , … McMurray, J. J. V. (2013). Clinical outcome endpoints in heart failure trials: A European Society of Cardiology Heart Failure Association consensus document. European Journal of Heart Failure, 15(10), 1082–1094. 23787718 10.1093/eurjhf/hft095

[bimj2016-bib-0035] Zanolla, L. , & Zardini, P. (2003). Selection of endpoints for heart failure clinical trials. European Journal of Heart Failure, 5(6), 717–723. 14675849 10.1016/s1388-9842(03)00101-6

[bimj2016-bib-0036] Zheng, S. L. , Chan, F. T. , Nabeebaccus, A. A. , Shah, A. M. , McDonagh, T. , Okonko, D. O. , & Ayis, S. (2018). Drug treatment effects on outcomes in heart failure with preserved ejection fraction: A systematic review and meta‐analysis. Heart, 104(5), 407–415. 28780577 10.1136/heartjnl-2017-311652 PMC5861385

[bimj2016-bib-0037] Zinman, B. , Wanner, C. , Lachin, J. M. , Fitchett, D. , Bluhmki, E. , Hantel, S. , … Inzucchi, S. E. (2015). Empagliflozin, cardiovascular outcomes, and mortality in type 2 diabetes. New England Journal of Medicine, 373(22), 2117–2128. 26378978 10.1056/NEJMoa1504720

